# Achievement of Diverse Domain Structures in Soft Magnetic Thin Film through Adjusting Intrinsic Magnetocrystalline Anisotropy

**DOI:** 10.1186/s11671-016-1789-7

**Published:** 2017-01-06

**Authors:** Juanying Jiao, Tao Wang, Tianyong Ma, Ying Wang, Fashen Li

**Affiliations:** Key Laboratory for Magnetism and Magnetic Materials of the Ministry of Education, Lanzhou University, Lanzhou, 730000 China

**Keywords:** Soft magnetic film, Domain structure, Magnetocrystalline anisotropy

## Abstract

Oriented soft magnetic hcp-Co_1 − *x*_Ir_*x*_ films with a fixed thickness of 120 nm were fabricated. All prepared films exhibit soft magnetic properties but various magnetocrystalline anisotropies with the variation of Ir content. The measured data shows that diverse domain structures including the Néel wall, Bloch wall, and stripe domains present in a fixed film thickness. It is singular for the single-layer soft magnetic film to possess diverse domains in a fixed thickness. This phenomenon was explained by introducing intrinsic magnetocrystalline anisotropy energy into soft magnetic films rather than the structural parameters of the film, inner stress, and microstructure effect.

## Background

In past decades, soft magnetic films have been applied in information storage technology and sensors. Their potential applications in the high-frequency area, such as inductor, noise suppressor, and field generation layer inside a spin torque oscillator, further spur researchers to investigate the moment distribution and property manipulation of soft magnetic thin films. In soft magnetic thin films [1–4], their moment distribution and macroscopic properties are strongly affected by the domain structure and the domain structure even determines their practical applications [5, 6]. In perpendicular magnetic recording, the existence of the Bloch wall in a soft magnetic underlayer causes the appearance of an out-of-plane stray field [7]. This feature leads to an additional noise signal during information reading. In the film which needs a rotatable anisotropy, the formation of a stripe domain structure is necessary [8, 9]. For a uniform soft magnetic single layer with in-plane isotropy, the domain structure is determined by competition of the exchange energy, magnetostatic energy, and magnetic anisotropy energies which are composed of magnetocrystalline anisotropy energy and stress-induced anisotropy energy. Generally, the magnetocrystalline anisotropy energy in traditional soft magnetic films can be neglected due to the weak magnetocrystalline anisotropy of grains. For this kind of soft magnetic films, all magnetic moments strictly lie in the film plane and the domain structure is formed through the Néel wall when the film thickness is small. The Néel wall will transform into the Bloch wall as the film thickness exceeds a critical value. The critical value of the Fe- and Co-based soft magnetic film with negligible magnetocrystalline anisotropy is approximately 20–40 nm [10–12]. When the film thickness is large enough, the stripe domain in which nearly all magnetic moments deviate from the film plane appears due to the weak perpendicular magnetic anisotropy originating from inner stress or a microstructure effect [13, 14].

Besides the film thickness, the domain structure can also be affected by the substrate and sputtering conditions which determine the inner stress and overall microstructure in magnetic layers and hence determine the perpendicular anisotropy which is the important origin for the stripe domain structure. According to reported literatures, the perpendicular anisotropy originating from the inner stress and microstructure effect in soft magnetic layers is usually very weak. When the film thickness is fixed, the accomplishment of dramatic variation of domain structures by changing the microstructure or stress without external manipulation is difficult. If the domain structure in soft magnetic layers can be changed easily through intrinsic magnetic parameters, the manipulation of domain structures will become easier and the expected domain structure can also exist in a fixed film thickness. This can promote the application of soft magnetic films in a wider area. In previous researches, the method for introducing magnetocrystalline anisotropy into a soft magnetic CoIr alloy film with orientation growth has been reported [15, 16]. The magnetocrystalline anisotropy constant of the CoIr alloy can even change from positive to negative with the variation of the Ir content [15]. In this study, the manipulation of the domain structure in soft magnetic CoIr films with a fixed film thickness was deeply investigated by adjusting the intrinsic magnetocrystalline anisotropy.

## Methods

**Fig. 1:**
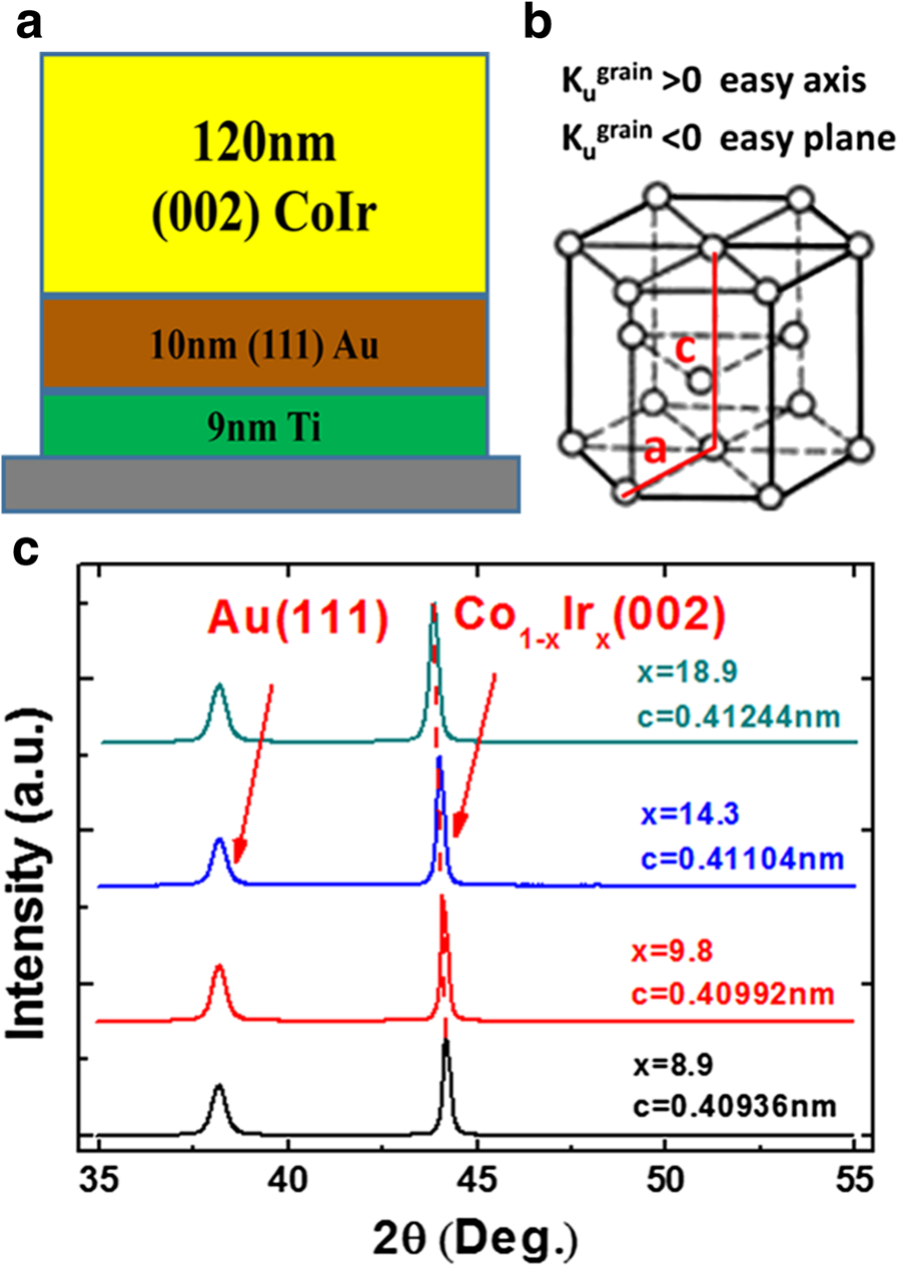
**a** Layer structure of the sputtered films with Ti (9 nm)/Au (10 nm)/Co_1 − *x*_Ir_*x*_ (120 nm). **b** Crystal structure of CoIr alloy. **c** XRD patterns of the oriented Co_1 − *x*_Ir_*x*_ films

Oriented hcp-Co_1 − *x*_Ir_*x*_ films were fabricated on Si wafers with surface oxidation by magnetron sputtering. The layer structure of the samples, which is shown in Fig. 1a, is substrate/Ti (9 nm)/Au (10 nm)/Co_1 − *x*_Ir_*x*_ (120 nm). The Ti layer was used to enhance adhesiveness of the Au seed layer and to induce the (111) orientation of the Au layer [17], and the Au seed layer was used to obtain the oriented hcp-CoIr alloy with its *c* axis perpendicular to the film plane. The pure Co with small Ir pieces on its surface was used as the target. The concentration of Ir can be manipulated by the number of Ir pieces to change the magnetocrystalline anisotropy of the CoIr grain. The thickness of all magnetic Co_1 − *x*_Ir_*x*_ layers was fixed as 120 nm. The sputter pressure was 0.25 and 0.30 Pa for the seed and CoIr layers, respectively. The crystal structure of the prepared films was analyzed by X-ray diffraction (XRD) with CuKα1 radiation (source wavelength is 1.5406 Å). A vibrating sample magnetometer (VSM) was used to measure their static magnetic properties and determine the magnetocrystalline anisotropy field of the CoIr alloy. A magnetic force microscope (MFM) was used to distinguish the domain structure of the prepared magnetic layers. Chemical composition of the magnetic layers was measured by an energy dispersive spectrometer (EDS).

## Results and Discussion

Figure 1b is a hexagonal crystalline system. The magnetocrystalline anisotropy energy of this system can be expressed by

1 $$ {E}_{\mathrm{grain}}={K}_1 \sin {}^2\theta +{K}_2 \sin {}^4\theta +{K}_3 \sin {}^6\theta +{K}_4 \sin {}^6\theta \cos 6\varphi $$

Here, the coefficients with a twofold symmetry term are summarized as ${K}_u^{\mathrm{grain}}$, and *K*_4_ is negligible due to that it is usually very small in this system. When ${K}_u^{\mathrm{grain}}$ is positive, the easy magnetization direction is along the *c* axis. This easy axis turns into a hard axis, and the magnetic moments prefer to lie in the *c* plane when ${K}_u^{\mathrm{grain}}$ is negative.

Figure 1c is the XRD patterns of the Co_1 − *x*_Ir_*x*_ films grown on the Au seed layer, which is measured by the *θ*–2*θ* scan method. Only two diffraction peaks are observed in a film. The peak at the lower angle is the (111) plane for Au. The strong peak at the higher angle corresponds to the (002) plane of the hcp-CoIr alloy due to that the hcp-Co alloy prefers to grow with its (002) plane on the (111) plane of Au [17]. This unique strong peak of the (002) plane reveals that the *c* crystal plane of Co_1 − *x*_Ir_*x*_ is parallel to the film plane. As the atomic diameter of Ir is bigger than that of Co, the lattice constant of the CoIr becomes smaller with the decrease of Ir content. This is verified by the left drift of the diffraction angle.

**Fig. 2:**
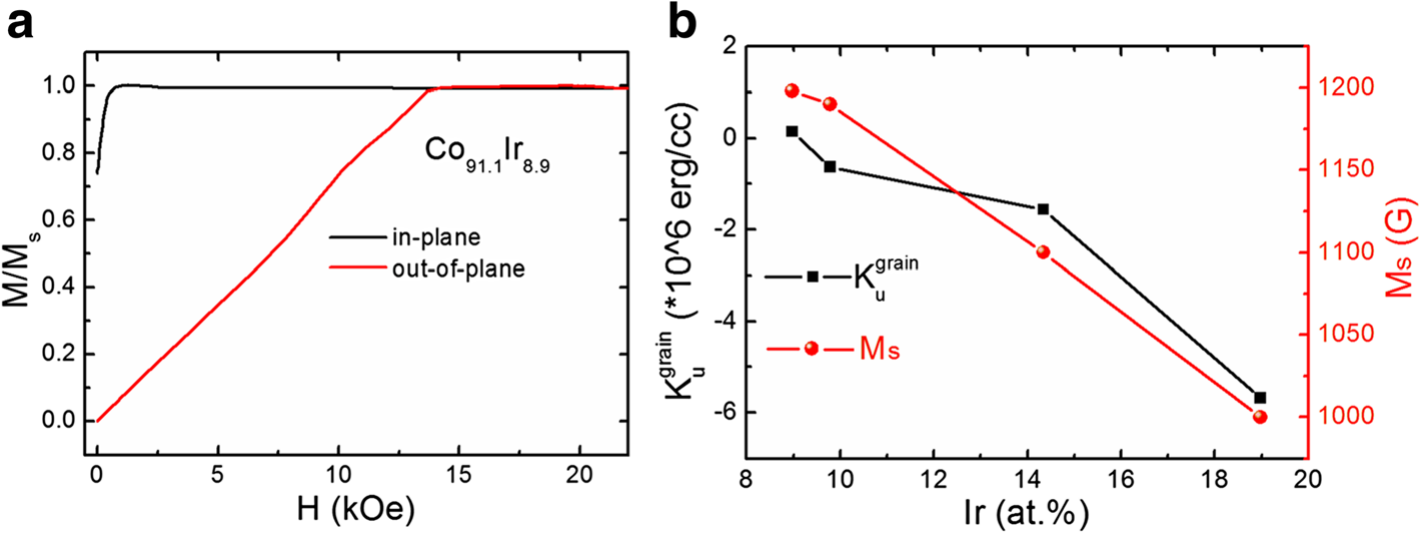
**a** Initial magnetization curves of the Co_91.1_Ir_8.9_ film parallel and perpendicular to the film plane. **b** Extracted magnetocrystalline anisotropy constant of the Co_1 − *x*_Ir_*x*_ alloy

To get the magnetocrystalline anisotropy constant ${K}_u^{\mathrm{grain}}$ of the Co_1 − *x*_Ir_*x*_ films with various Ir content, the initial magnetization curves parallel and perpendicular to the film plane were measured. Figure 2a shows typical results of the Co_91.1_Ir_8.9_ film. The total out-of-plane anisotropy field *H*_out_ can be obtained by calculating the area between two magnetization curves. The *H*_out_ should be composed of demagnetization field 4π*M*_*s*_ and magnetocrystalline anisotropy field ${K}_u^{\mathrm{grain}}$. The demagnetization field can be obtained from the measured saturation magnetization shown in Fig. 2b. The ${K}_u^{\mathrm{grain}}$ can be gotten by − [(*H*_out_ − 4*πM*_*s*_)*M*_*s*_]/2. When *H*_out_ is larger than 4π*M*_*s*_, ${K}_u^{\mathrm{grain}}$ is negative; on the contrary, ${K}_u^{\mathrm{grain}}$ is positive. Figure 2b shows the calculated ${K}_u^{\mathrm{grain}}$ values from the measured data. When *x* = 18.9, the film has the strongest magnetocrystalline anisotropy with a negative ${K}_u^{\mathrm{grain}}$. As *x* decreases, the magnetocrystalline anisotropy becomes weak while ${K}_u^{\mathrm{grain}}$ keeps the negative value. When *x* = 8.9, ${K}_u^{\mathrm{grain}}$ turns to be a small positive value. The positive ${K}_u^{\mathrm{grain}}$ indicates the *c* axis in the CoIr alloy is the easy axis. As the *c* axis of the CoIr alloy grows perpendicular to the film plane, the positive ${K}_u^{\mathrm{grain}}$ results in a perpendicular magnetic anisotropy of Co_91.1_Ir_8.9_ in the film.

**Fig. 3:**
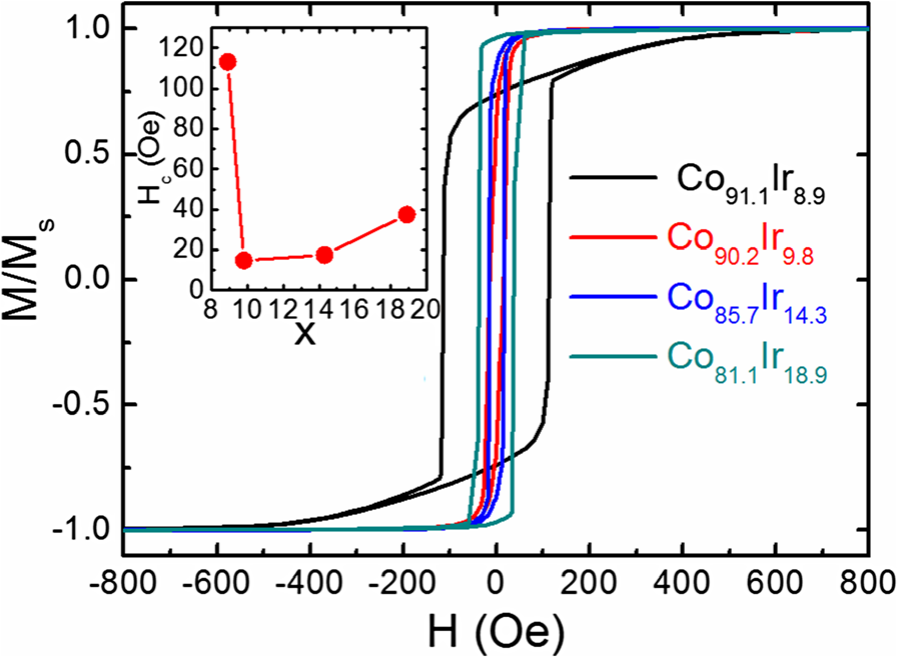
Room temperature of the in-plane hysteresis loops of the samples

Figure 3 gives the in-plane hysteresis loops of the films measured at room temperature. The inset shows the coercivities of the samples. For the films with negative ${K}_u^{\mathrm{grain}}$, the coercivity and applied magnetic field required for saturation are all small. When ${K}_u^{\mathrm{grain}}$ is negative, the *c* crystal plane of CoIr alloy is the easy magnetization plane and *K*_4_ is very small in this system. As the fabricated films are composed of oriented CoIr grains with its *c* crystal plane parallel to the film plane, this leads to good soft magnetic properties along the film plane although the magnetocrystalline anisotropy of the film is strong. Moreover, in this situation, both the demagnetization field and magnetocrystalline anisotropy field spur magnetic moments to lie in the film plane. When *x* = 8.9, the sample exhibits quite different properties. The magnetization process appears to be a transcritical hysteresis loop, which is a strong signature of the formation of stripe domain structures [8, 9, 14]. It is speculated that the perpendicular magnetic anisotropy originating from positive ${K}_u^{\mathrm{grain}}$ of the CoIr alloy supports the formation of stripe domain structures. It should be noticed that the coercivity in the film with stripe domain is larger compared with that of the films without stripe domain structures. This result is quite consistent with other reports on films with stripe domains and can be interpreted in terms of rotatable anisotropy often found in thin films with stripe domains [8, 9, 14, 18].

**Fig. 4:**
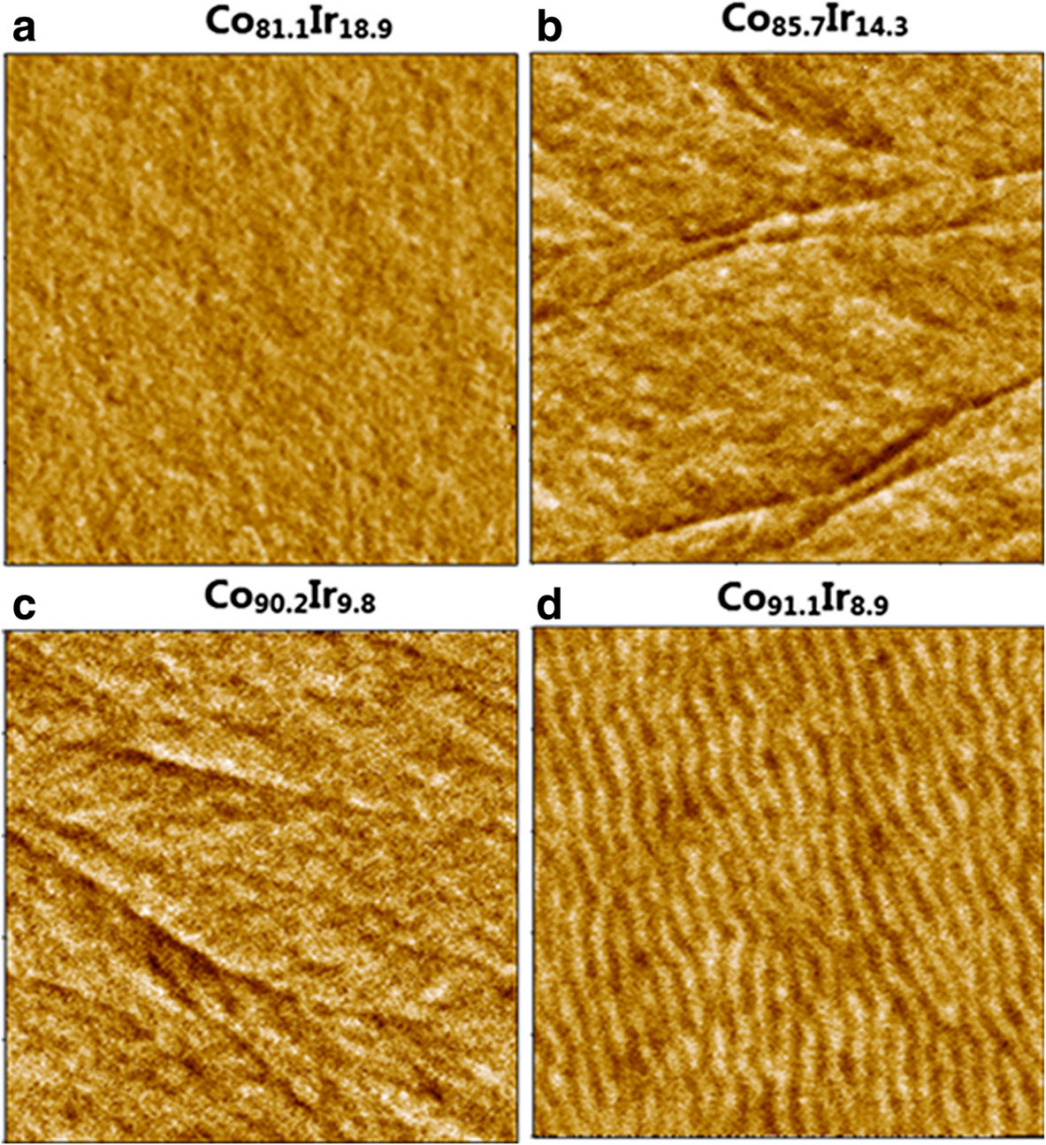
MFM pattern of Co_1-x_Ir_x_ films with a scanning area of 5μm×5μm. **a** out-of-plane stray field is not detected for Co_81.1_Ir_18.9_; (**b**)-(**c**) out-of-plane stray field in transition area of magnetic moment reversal is detected for Co_85.7_Ir_14.3_ and Co_90.2_Ir_9.8_, respectively (**d**) a stripe domain structure is clearly shown with bright and black stripes for Co_91.1_Ir_8.9_

**Fig. 5:**
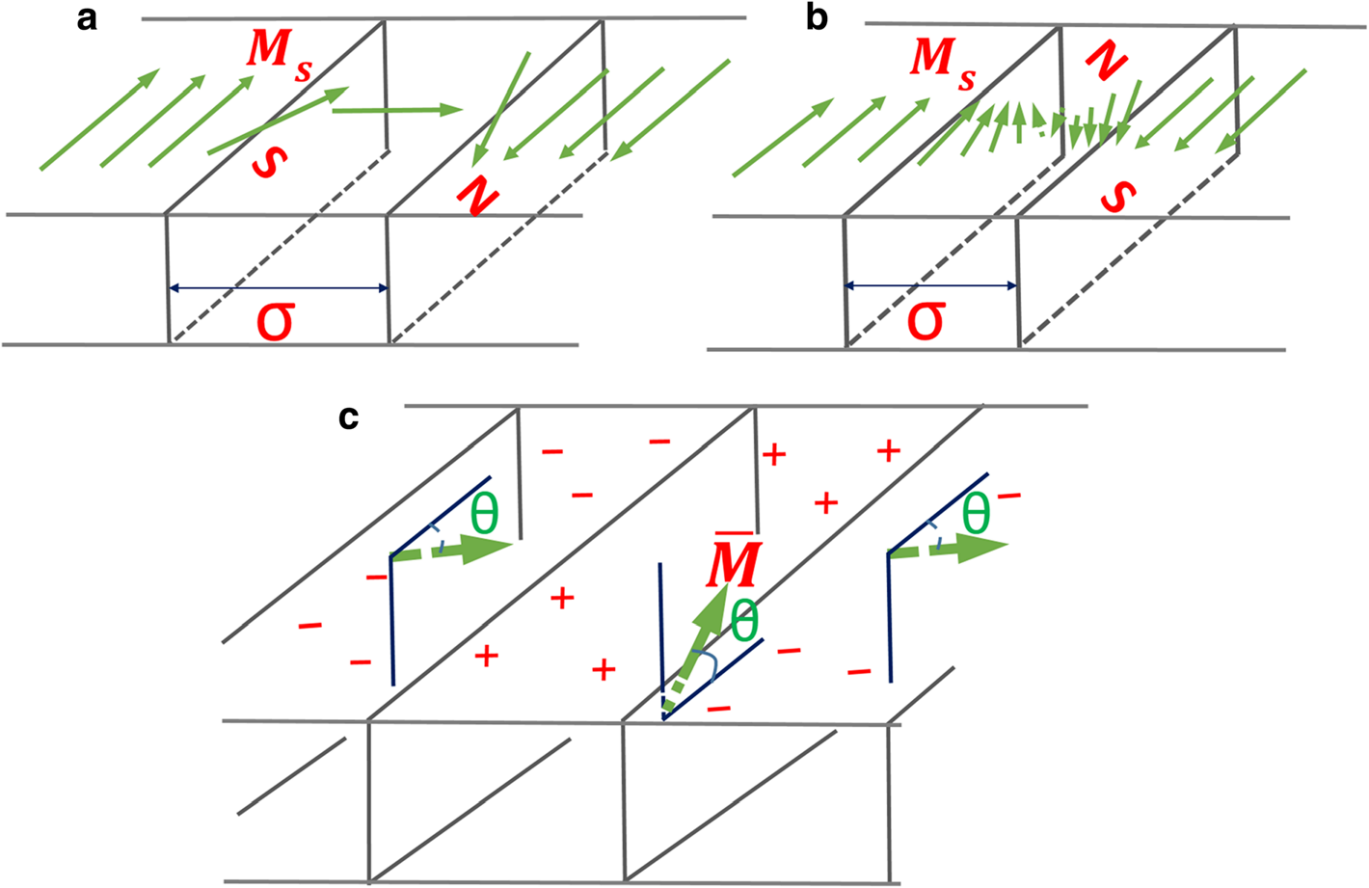
The schematics of the domain structure. **a** Néel wall. **b** Bloch wall. **c** Stripe domain structure

**Fig. 6:**
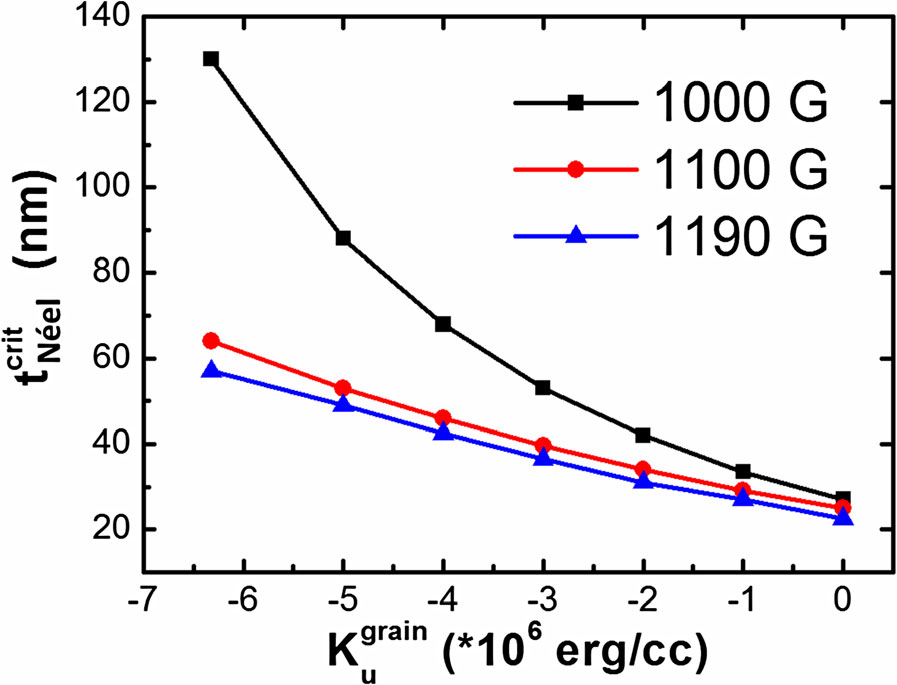
The dependence of the critical transition thickness from the Néel wall to the Bloch wall on ${K}_u^{\mathrm{grain}}$ at *M*_*s*_ = 1000, 1100, and 1190 G

Figure 4 displays the MFM images of the films in demagnetization state with an area of 5 μm × 5 μm. For the Co_1 − *x*_Ir_*x*_ film with *x* = 18.9, any out-of-plane stray field is not detected. All magnetic moments should strictly lie in the film plane, and the domain structure should be formed through the Néel wall. The schematic of the domain structure was shown in Fig. 5a. Generally, the transition thickness of the magnetic layer from the Néel wall to the Bloch wall for Fe- and Co-based soft magnetic film with negligible magnetocrystalline anisotropy is about 20–40 nm. However, the oriented soft magnetic Co_91.1_Ir_8.9_ film with a thickness of 120 nm still possesses a Néel wall. This is due to the enhanced transition thickness from the Néel wall to the Bloch wall, which can be explained by the following equations for the Néel wall energy *E*_Néel_ and the Bloch wall energy *E*_Bloch_ [15]:

2 $$ {E}_{\mathrm{N}\acute{\mathrm{e}} \mathrm{el}}={A}_1\frac{\pi^2}{\sigma }+\frac{\pi {M_s}^2{\sigma}^2}{t+\sigma } $$

3 $$ {E}_{\mathrm{Bloch}}={A}_1\frac{\pi^2}{\sigma }+\frac{-{K}_u^{\mathrm{grain}}}{2}\sigma +\frac{\pi {M_s}^2t{\sigma}^2}{t+\sigma } $$

where *t*, *σ*, and *A*_1_ denote magnetic layer thickness, domain wall thickness, and exchange stiffness constant, respectively. The type of domain wall depends on which is smaller for *E*_Néel_ and *E*_Bloch_. The critical transition thickness of the magnetic layer from the Néel wall to the Bloch wall can be achieved when *E*_Néel_ = *E*_Bloch_. The dependence of the critical transition thickness from the Néel wall to the Bloch wall on ${K}_u^{\mathrm{grain}}$ by using *M*_*s*_ of Co_81.1_Ir_18.9_ (1000 G) and the exchange stiffness constant of pure cobalt are shown in Fig. 6. The critical thickness increases with the increase of $\left|{K}_u^{\mathrm{grain}}\right|$. For the oriented Co_81.1_Ir_18.9_ film, its ${K}_u^{\mathrm{grain}}=-6.3\times {10}^6\mathrm{erg}/\mathrm{cc}$. The calculated transition thickness is 130 nm. Hence, the Co_81.1_Ir_18.9_ film with a thickness of 120 nm still possesses the Néel wall. When *x* = 14.3 and 9.8, the magnetocrystalline anisotropy of the films becomes weaker compared with the Co_81.1_Ir_18.9_ film and the domain wall characterized by black and white lines in the MFM images is obviously detected. This domain wall should be a Bloch wall due to that the out-of-plane stray field in the transition area of the magnetic moment reversal is detected by MFM and the magnetic moments except the domain wall area still lie in the film plane. The schematic of the domain structure was shown in Fig. 5b. Figure 6 also shows the critical thickness dependence of ${K}_u^{\mathrm{grain}}$ by using *M*_*s*_ of Co_85.7_Ir_14.3_ (1100 G) and Co_90.2_Ir_9.8_ (1190 G). The calculated transition thicknesses are only 38 and 28 nm for the Co_85.7_Ir_14.3_ and Co_90.2_Ir_9.8_ films, respectively. As the actual film thickness is much larger than the transition thicknesses, the Bloch wall should exist.

When ${K}_u^{\mathrm{grain}}$ turns to be positive, the film with *x* = 8.9 clearly exhibits a stripe domain structure (Fig. 5c displays its schematic), due to that the MFM image is composed of bright and black stripes. This MFM image is consistent with the transcritical hysteresis loop in Fig. 3 which is a signature of the formation of the stripe domain structure. According to the reported literatures, the film thickness possessing the stripe domain is about several hundred nanometers and the stripe domain comes from the weak perpendicular magnetic anisotropy that resulted from a stain and microstructure effect [8, 13, 19–22]. Its critical thickness can be described by

4 $$ {t}_{\mathrm{stripe}}^{\mathrm{crit}}=2\pi \sqrt{A/{K}^{\mathrm{perp}}} $$

*K* ^perp^ originating from stress or a microstructure effect is generally very weak. This leads to a larger ${t}_{\mathrm{stripe}}^{\mathrm{crit}}$ from the above equation. In this study, the oriented Co_91.1_Ir_8.9_ thin film exhibits the stripe domain structure in a relatively small thickness. In the oriented Co_91.1_Ir_8.9_ thin film, there is the uniaxial magnetocrystalline anisotropy ${K}_u^{\mathrm{grain}}$ perpendicular to the film plane. This ${K}_u^{\mathrm{grain}}$ has the same effect as *K* ^perp^ on the formation of the stripe domain structure. Moreover, the positive ${K}_u^{\mathrm{grain}}$ can be manipulated easily by varying the Ir content, so the ${t}_{\mathrm{stripe}}^{\mathrm{crit}}$ can also be adjusted easily in this film system.

## Conclusions

In summary, the diverse domain structures in oriented soft magnetic Co_1 − *x*_Ir_*x*_ thin films with a fixed thickness were reported. The magnetocrystalline anisotropy energy was introduced into total energy in soft magnetic films to adjust the domain structure. When ${K}_u^{\mathrm{grain}}$ varies from negative to positive, the domain structure exhibits a diverse change from the Néel wall domain to the Bloch wall domain and then to the stripe domain with all magnetic moments deviating from the film plane. All these changes are accomplished only by adjusting the intrinsic magnetic parameters. This work offers a new avenue to manipulate the domain structure in single-layer soft magnetic films except film thickness, inner stress, and microstructure determined by sputtering conditions.

## Abbreviations

EDS: Energy dispersive spectrometer
MFM: Magnetic force microscope
VSM: Vibrating sample magnetometer
XRD: X-ray diffraction

## References

[1] Greve H, Pochstein C, Takele H, Zaporojtchenko V, Faupel F, Gerber A, Frommberger M, Quandt E (2006). Nanostructured magnetic Fe–Ni–Co/Teflon multilayers for high-frequency applications in the gigahertz range. Appl Phys Lett.

[2] Yutaka Shimada JM, Tetsuo I, Kunio Y, Yasushi E, Sho M, Masahiro Y (2014). Performance of crossed anisotropy multilayered cozrnb films as IC Chip Level Electromagnetic noise suppressor. IEEE Trans Magnetics.

[3] Sohn J, Han SH, Yamaguchi M, Lim SH (2007). Si-based electromagnetic noise suppressors integrated with a magnetic thin film. Appl Phys Lett.

[4] Nozawa N, Saito S, Kimura T, Shibuya K, Hoshino K, Hinata S, Takahashi M (2013). Giant negative uniaxial magnetocrystalline anisotropy of Co80Ir20 sputtered films with perfect hexagonal-close-packed and composition-modulated atomic layer stacking. Appl Phys Lett.

[5] Cooper EI, Te CB, Heidmann J, Hsu Y, Kern P, Lam JW, Ramasubramanian M, Robertson N, Romankiw LT, Xu H (2005). Recent developments in high-moment electroplated materials for recording heads. IBM J Res Dev.

[6] Greve H, Woltermann E, Quenzer H-J, Wagner B, Quandt E (2010). Giant magnetoelectric coefficients in (Fe90Co10) 78 Si 12 B 10-AIN thin film composites. Appl Phys Lett.

[7] Park S, Zhu J-G, Laughlin DE (2009) A novel crystalline soft magnetic intermediate layer for perpendicular recording media. J Appl Phys 105:07B723

[8] Chai GZ, Phuoc NN, Ong CK (2013). High thermal stability of zero-field ferromagnetic resonance above 5 GHz in ferrite-doped CoFe thin films. Appl Phys Lett.

[9] Fin S, Tomasello R, Bisero D, Marangolo M, Sacchi M, Popescu H, Eddrief M, Hepburn C, Finocchio G, Carpentieri M, Rettori A, Pini MG, Tacchi S (2015). In-plane rotation of magnetic stripe domains in Fe1−xGax thin films. Phys Rev B.

[10] Liou SH, Sabiryanov RF, Jaswal SS, Wu J, Yao YD (2001). Magnetic domain patterns of rectangular and elliptic arrays of small permalloy elements. J Magn Magn Mater.

[11] Chechenin NG, Chezan AR, Craus CB, Vystavel T, Boerma DO, de Hosson J, Th M, Niesen L (2002). Microstructure of nanocrystalline FeZr(N)-films and their soft magnetic properties. J Magn Magn Mater.

[12] Svalov AV, Aseguinolaza IR, Garcia-Arribas A, Orue I, Barandiaran JM, Alonso J, Fernández-Gubieda ML, Kurlyandskaya GV (2010). Structure and magnetic properties of thin permalloy films Near the “Transcritical” state. IEEE Trans Magnetics.

[13] Amos N, Fernandez R, Ikkawi R, Lee B, Lavrenov A, Krichevsky A, Litvinov D, Khizroev S (2008) Magnetic force microscopy study of magnetic stripe domains in sputter deposited Permalloy thin films. J Appl Phys 103:07E732

[14] Coïsson M, Celegato F, Olivetti E, Tiberto P, Vinai F, Baricco M (2008). Stripe domains and spin reorientation transition in Fe78 B13 Si9 thin films produced by rf sputtering. J Appl Phys.

[15] Hashimoto A, Saito S, Takahashi M (2006) A soft magnetic underlayer with negative uniaxial magnetocrystalline anisotropy for suppression of spike noise and wide adjacent track erasure in perpendicular recording media. J Appl Phys 99:08Q907

[16] Xu F, Zhang S, Yang D, Wang T, Li F (2015) High-frequency properties of oriented hcp-Co1−xIrx (0.06 ≤ x ≤ 0.24) soft magnetic films. J Appl Phys 117:17B725

[17] Kondo Y, Ariake J, Chiba T, Taguchi K, Suzuki M, Kawamura N, Honda N (2011). Investigation on the origin of switching field width in Co-Pt dot array. Phys Procedia.

[18] Wang G, Dong C, Wang W, Wang Z, Chai G, Jiang C, Xue D (2012). Observation of rotatable stripe domain in permalloy films with oblique sputtering. J Appl Phys.

[19] Phuoc NN, Ong CK (2013). Thermal stability of high frequency properties of gradient-composition-sputtered FeCoHf films with and without stripe domains. J Appl Phys.

[20] McCord J, Erkartal B, von Hofe T, Kienle L, Quandt E, Roshchupkina O, Grenzer J (2013) Revisiting magnetic stripe domains—anisotropy gradient and stripe asymmetry. J Appl Phys 113:073903

[21] Basith MA, McVitie S, Strache T, Fritzsche M, Muecklich A, Fassbender J, McCord J (2015). Lorentz TEM imaging of stripe structures embedded in a soft magnetic matrix. Phys Rev Applied.

[22] Samantaray B, Singh AK, Perumal A, Ranganathan R, Mandal P (2015). Spin dynamics and frequency dependence of magnetic damping study in soft ferromagnetic TeTaC film with a stripe domain structure. AIP Adv.

